# DNA palette code for time-series archival data storage

**DOI:** 10.1093/nsr/nwae321

**Published:** 2024-09-10

**Authors:** Zihui Yan, Haoran Zhang, Boyuan Lu, Tong Han, Xiaoguang Tong, Yingjin Yuan

**Affiliations:** Frontiers Science Center for Synthetic Biology and Key Laboratory of Systems Bioengineering (Ministry of Education), School of Chemical Engineering and Technology, Tianjin University, Tianjin 300072, China; Frontiers Research Institute for Synthetic Biology, Tianjin University, Tianjin 300072, China; Frontiers Science Center for Synthetic Biology and Key Laboratory of Systems Bioengineering (Ministry of Education), School of Chemical Engineering and Technology, Tianjin University, Tianjin 300072, China; Frontiers Research Institute for Synthetic Biology, Tianjin University, Tianjin 300072, China; Frontiers Science Center for Synthetic Biology and Key Laboratory of Systems Bioengineering (Ministry of Education), School of Chemical Engineering and Technology, Tianjin University, Tianjin 300072, China; Frontiers Research Institute for Synthetic Biology, Tianjin University, Tianjin 300072, China; Department of Neurosurgery, Huanhu Hospital, Tianjin 300350, China; Department of Neurosurgery, Huanhu Hospital, Tianjin 300350, China; Frontiers Science Center for Synthetic Biology and Key Laboratory of Systems Bioengineering (Ministry of Education), School of Chemical Engineering and Technology, Tianjin University, Tianjin 300072, China; Frontiers Research Institute for Synthetic Biology, Tianjin University, Tianjin 300072, China

**Keywords:** DNA data storage, synthetic biology, medical imaging, error-correcting codes

## Abstract

The long-term preservation of large volumes of infrequently accessed cold data poses challenges to the storage community. Deoxyribonucleic acid (DNA) is considered a promising solution due to its inherent physical stability and significant storage density. The information density and decoding sequence coverage are two important metrics that influence the efficiency of DNA data storage. In this study, we propose a novel coding scheme called the DNA palette code, which is suitable for cold data, especially time-series archival datasets. These datasets are not frequently accessed, but require reliable long-term storage for retrospective research. The DNA palette code employs unordered combinations of index-free oligonucleotides to represent binary information. It can achieve high net information density encoding and lossless decoding with low sequencing coverage. When sequencing reads are corrupted, it can still effectively recover partial information, preventing the complete failure of file retrieval. The *in vitro* testing of clinical brain magnetic resonance imaging (MRI) data storage, as well as simulation validations using large-scale public MRI datasets (10 GB), planetary science datasets and meteorological datasets, demonstrates the advantages of our coding scheme, including high net information density, low decoding sequence coverage and wide applicability.

## Teaser

This work introduces the DNA Palette code, a novel coding scheme designed for efficient DNA data storage of time-series archival datasets, validated through in vivo experiments and large-scale simulations.

## INTRODUCTION

Deoxyribonucleic acid (DNA) has recently received significant attention as a promising candidate for data storage media owing to its extended lifespan and inherent storage density [1–4], especially for cold data, which refers to data with low access frequency and reading speed requirements, but with large volumes that need to be stored and managed for the long term [5]. Specifically, examples include three-dimensional medical imaging data [6], planetary science data monitoring changes in planetary states [7] and meteorological data documenting weather fluctuations [8]. These datasets typically require large-scale data volumes and persistent archiving for historical trend analysis and retrospective research, resulting in high storage costs when stored using conventional storage media. By examining the characteristics of such datasets, we observed that they commonly consist of multiple files generated at different time points, sharing the same format, and containing inherent continuous content. In the context of this study, we refer to them as time-series archival datasets.

Besides, strict scientific standards require high accuracy in the recovery of such data. However, due to the unpredictability of biochemical reactions during the synthesis, manipulation and sequencing processes, the DNA data storage process is error prone [9,10]. To enhance the reliability of DNA data storage, various concatenated codes have been proposed. In the process of inferring the order of disordered and duplicate DNA sequencing reads, the outer code typically assigns a unique index to each encoded oligonucleotide (oligo), enabling the identification of its location. Consequently, dropout errors can be treated as erasure errors. Erasure codes, such as the DNA fountain code [11–13], indexed Reed–Solomon (RS) code [14,15] and Low-density Parity-check (LDPC) code [16], are proven effective in restoring the missing information. We refer to them as index-added encoding strategies. Meanwhile, several error-correcting codes, including the watermarker code [17], RS code [11,15], HEDGES code [14], DNA-Aeon code [13], Derrick code [18] and SPIDER-WEB code [19] are considered valid inner codes for checking and correcting nucleotide errors.

While numerous studies have proposed many coding schemes for DNA data storage, there is still potential for designing an encoding method that better adapts to time-series archival datasets. Moreover, most studies compress the raw information before encoding, which can lead to complete failure of data recovery even with a minor error. This phenomenon has been found in many studies [16,20,21]. Current compression algorithms are not tailored for DNA data storage and are not suitable for scenarios with high error rates. It inspires us not to use compression algorithms, but to focus on the characteristics of the raw information itself, so as to design an adaptive encoding method to improve information density and correct errors.

To achieve this goal, considering the characteristics of time-series archival datasets, we proposed a novel coding method called the DNA palette code. The main features of our coding scheme are that it does not require indexing, and can achieve high information density (i.e. the ratio of input bit information to the number of synthetic DNA nucleotides, excluding primers and adapters [11]) and a low decoding sequence coverage rate (i.e. the number of reads required to recover 100$\%$ information divided by the number of encoded oligos). In scenarios where sequencing coverage is very low, leading to high dropout rates and byte error rates, the decoder is still capable of recovering partial information. Our coding scheme is resilient to residual byte errors, allowing it to recover partial information to prevent complete data loss even in the presence of such errors. We verified the performance of the DNA palette code by simulation and experimental validation. In our *in vitro* test, we encoded 11.28 MB of clinical brain magnetic resonance imaging (MRI) data into 255 248 oligos of 155-nt length (data payload only, no primers and adapters). The information was successfully recovered with 100$\%$ accuracy at a median average coverage of 4.4$\times$. When the sequencing coverage is 2$\times$, the recovered pixel data can also provide medical information. Furthermore, we conducted simulations on a large public MRI dataset (10 GB) and two other applications (including observations of the Earth’s plasmasphere by the extreme ultraviolet camera on the Chang’E-3 Moon lander and daily melt results on the surface of the Greenland ice sheet). This illustrates the robustness and broad application of the DNA palette code.

## RESULTS

**Figure 1.**
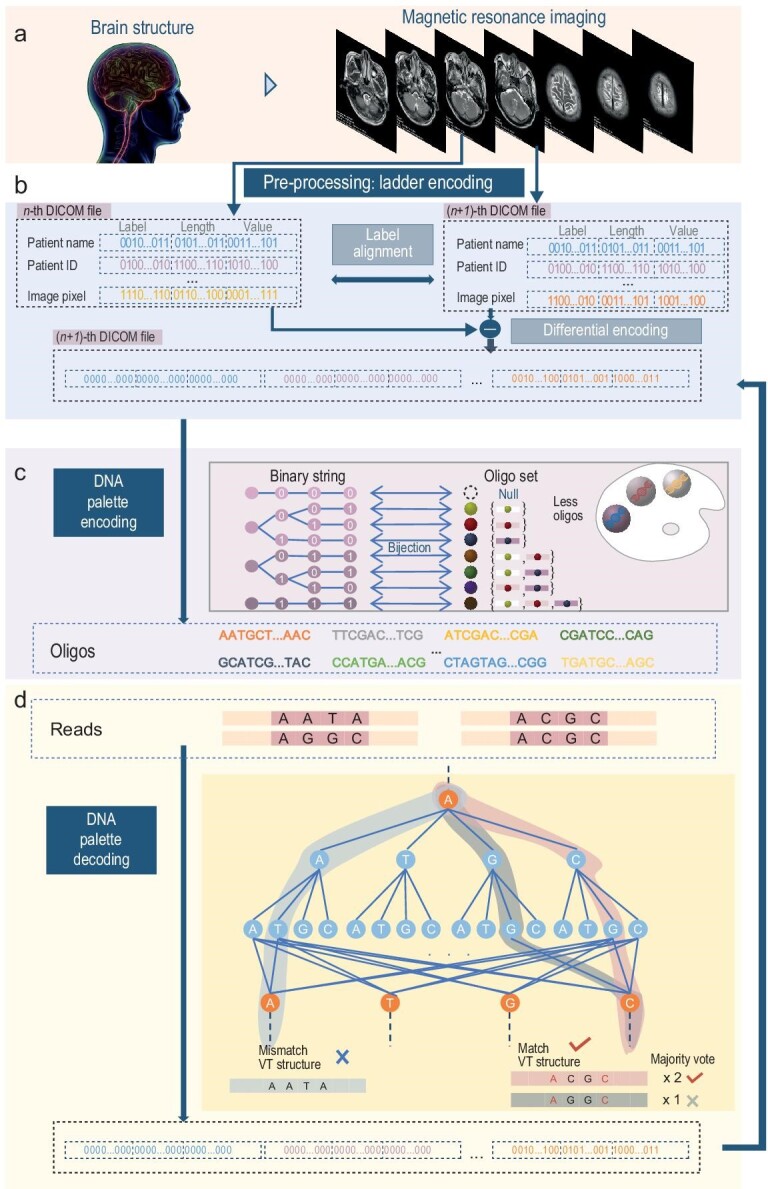
Overview of the DNA palette coding scheme. (a) Primary application: brain MRI scan. A brain MRI scan is a diagnostic test that produces clear images of the internal structures of the brain, generating DICOM files across multiple slices. (b) Data pre-processing process: DNA ladder code. The DNA ladder code involves label alignment, differential encoding and RS encoding. (c) Exemplification of simplified DNA palette encoding. The DNA palette code converts a binary sequence into precisely one set of oligos with a quaternary Varshamov-Tenengolts (VT) structure. (d) Illustration of DNA palette decoding. A read is considered correct only if it adheres to the VT structure and constitutes the majority of accurate reads with the same labels.

A brain MRI scan yields a significant volume of slice data, where each slice is stored as an individual digital imaging and communications in medicine (DICOM) file [22] (Fig. 1a). Adhering to the DICOM format (Fig. S1), we introduced a data pre-processing scheme based on dictionary transforms, called the DNA ladder code, which can utilize the structural information of the dataset to convert it into a form more conducive to the DNA palette code encoding (Fig. 1b). Subsequently, we presented a ‘bit-to-oligo’ mapping approach grounded in combinatorial theory, termed the DNA palette code (Fig. 1c). The decoder undertakes trace reconstruction and nucleotide error-correcting tasks, accommodating duplicate sequencing reads without the need for clustering or multiple alignments (Fig. 1d). The DNA palette code is the major innovation, so we use it to refer to the coding scheme proposed in this work.

### DNA palette code

The fundamental idea of the DNA palette code is to establish a bijection between $\mathcal {X}^N$, the range of the raw information, and $\mathcal {O}$, the family of oligo sets. Here, we use $\mathcal {X} = \lbrace 0,1\rbrace$ to denote the binary alphabet and $\mathcal {D} = \lbrace \text{A},\text{T},\text{G},\text{C}\rbrace$ to represent four natural DNA nucleobases: adenine (A), cytosine (C), guanine (G) and thymine (T). The codeword corresponding to $\boldsymbol{x} \in \mathcal {X}^n$ is a subset of the oligo set. Metaphorically, the DNA palette code regards each oligo as a pigment, distinguishing different binary strings by coloring them with distinct colors. It enables the mixing of different pigments to create new colors. Assuming that mixed colors are distinct, and pigments exhibit a total order relation, the resulting mixed colors will possess a lexicographical order defined by this relation. Similarly, we have also defined a total order on the range of the raw information. This allows us to establish a one-to-one mapping between binary strings and colors through these two total order relations. Consequently, the input can be uniquely encoded as a color. Since pigments are mixed without regard to the order or quantity added, the number of pigments used for encoding binary strings is not fixed and no index needs to be inserted for each pigment.

When considering oligos as pigments, the mixing process of pigments can be viewed as sampling without replacement within the set of oligos. Here is a straightforward example. Let ‘001’ be the raw information, and let $O = \lbrace \boldsymbol{o}_1, \boldsymbol{o}_2, \boldsymbol{o}_3\rbrace$ be the preset oligos. The range of the raw information is $\mathcal {X}^3 = \lbrace 000, 001, 010, 100, 011, 101, 110, 111\rbrace$, and the family of oligo sets is $\mathcal {O}^3 = \lbrace O_0, O_1, \ldots , O_7\rbrace$, where $O_0 = \emptyset$, $O_1 = \lbrace \boldsymbol{o}_1\rbrace$, $O_2 = \lbrace \boldsymbol{o}_2\rbrace$ and so on, up to $O_7 = \lbrace \boldsymbol{o}_1, \boldsymbol{o}_2, \boldsymbol{o}_3\rbrace$. We can define a map to encode sequences in $\mathcal {X}^3$ to oligo sets in $\mathcal {O}^3$, such as $000 \mapsto O_0$, $001 \mapsto O_1$ and so forth. The decoder can determine the raw information by identifying which oligo was received. Notably, the number of encoded oligos is not fixed due to $|O_0| \ne |O_1|$. This is a key feature of our encoding scheme, allowing fewer oligos/nucleotides than expected to represent the raw information. Specifically, using a typical transcoding method (e.g., 00 $\leftrightarrow$ A, 01 $\leftrightarrow$ T, $10 \leftrightarrow {\rm G}$, 11 $\leftrightarrow$ C), ‘001’ would be encoded as ‘AT’. In contrast, our method would encode ‘001’ into $O_1 = \lbrace \boldsymbol{o}_1\rbrace = \lbrace \text{A}\rbrace$ when $O = \lbrace \text{A, T, G}\rbrace$.

For a longer binary information sequence (i.e. $\boldsymbol{x}\in \mathcal {X}^N$) and a preset oligo set $O=\lbrace \boldsymbol{o}_1,\boldsymbol{o}_2,\ldots ,\boldsymbol{o}_n\rbrace \subseteq \mathcal {D}^m$ ($n> N$), we designed a map *f* to encode this binary information into an oligo set. Firstly, the order ‘$< $’ of these preset oligos is defined as follows: for any $\boldsymbol{o}_1 = (o_1^1, o_2^1, \ldots , o_m^1) \in \mathcal {D}^m$ and $\boldsymbol{o}_2 = (o_1^2, o_2^2, \ldots , o_m^2) \in \mathcal {D}^m$, if there exists *i* such that $o_1^1 = o_1^2, o_2^1 = o_2^2, \ldots , o_{i-1}^1 = o_{i-1}^2$ and $o_i^1 < o_i^2$ then $\boldsymbol{o}_1 < \boldsymbol{o}_2$. The order of nucleotides is ${\rm A} < {\rm T} < {\rm G} < {\rm C}$. For example, ${\rm AAT} < {\rm AAG} < {\rm ATT}$. It can be easily proved that this order is transitive, antisymmetric and strongly connected, thus constituting a total order on $\mathcal {D}^m$. Without loss of generality, we defined $\boldsymbol{o}_1< \boldsymbol{o}_2< \cdots < \boldsymbol{o}_n$. Then we defined a total order on $\mathcal {O}$, where $\mathcal {O}$ is the family of the subsets of *O*: for $A=\lbrace \boldsymbol{o}^{A}_1,\boldsymbol{o}^{A}_2,\ldots ,\boldsymbol{o}^{A}_{m}\rbrace \subseteq O$ and $B=\lbrace \boldsymbol{o}^{B}_1,\boldsymbol{o}^{B}_2,\ldots ,\boldsymbol{o}^{B}_s\rbrace \subseteq O$, (i) if there exists *j* such that $\boldsymbol{o}^{A}_m=\boldsymbol{o}^{B}_s$, $\boldsymbol{o}^{A}_{m-1}=\boldsymbol{o}^{B}_{s-1}$,...,$\boldsymbol{o}^{A}_{m-j+1} =\boldsymbol{o}^{B}_{s-j+1}$, $\boldsymbol{o}^{A}_{m-j}< \boldsymbol{o}^{B}_{s-j}$, then $A< B$; (ii) otherwise, $B< A$. Next, we defined a map $f:\mathcal {X}^N \rightarrow \mathcal {O}^N$ such that $f(\boldsymbol{x})$ is the $(\sum _{i=1}^N2^{i-1}x_i)$th set in $\mathcal {O}$. Here, $\mathcal {O}^N$ denotes the first $2^N$ sets of $\mathcal {O}$. It is easy to prove that *f* is a bijection. According to the map *f*, the raw binary information $\boldsymbol{x}$ will be encoded into $f(\boldsymbol{x})$. The decoder can recover the raw information via $f^{-1}$, where $f^{-1}$ is determined when given *O* and *N*.

The above encoding scheme requires that both the encoder and decoder know the one-to-one mapping alphabet. This may pose a challenge when storing large-scale datasets. We propose a nested mapping method, as shown in Fig. 1b and Fig. S2. This method splits the large mapping into a cascade of two small mappings. The details of the nested encoding scheme are shown in Section S1.1 within the online supplementary material. Besides, In practice, we adopted the nested mapping and presented the encoded oligos to satisfy the VT structure [23–25].

The decoding algorithm of DNA palette code can be regarded as a trace reconstruction process aimed at recovering the correct set of oligos from duplicate sequencing reads (Fig. 1d). Specifically, each read undergoes an initial check for compliance with the VT structure, encompassing error-correcting and verification processes. Subsequently, the reads are grouped and selected by the majority vote algorithm. Following these two steps, a substantial portion of the encoded oligos can be successfully restored. Notably, our decoder takes duplicate sequencing reads as input, rather than using the central sequence obtained from clustering or multiple sequence alignment algorithms as input. The decoding complexity of the majority vote algorithm scales linearly with the number of sequencing reads. Additionally, in adherence to the total order sorting rules, bits/oligos can be directly placed in their respective positions without necessitating the comparison and exchange process. This results in linear encoding and decoding complexity relative to the number of bits/oligos.

Upon recalling the encoding process of the DNA palette code, it is noted that the number of encoded oligos is related to the Hamming weight of the raw binary information. To this end, we proposed a data pre-processing method called the DNA ladder code to convert the raw information into a new form containing a large number of zeros. This algorithm encompasses three stages: label alignment, differential encoding and block RS encoding. The initial two stages modify the structure of the raw information without introducing redundancy, while the third stage incorporates parity-check bits to address errors. Specifically, residual errors in the DNA palette decoder and the occurrence of dropout oligos might lead to a partial loss of information in the decoded binary string, which can be recovered through the RS code. A comprehensive description of the DNA ladder code is available in Section S1.3.

### Testing *in vitro*

**Figure 2.**
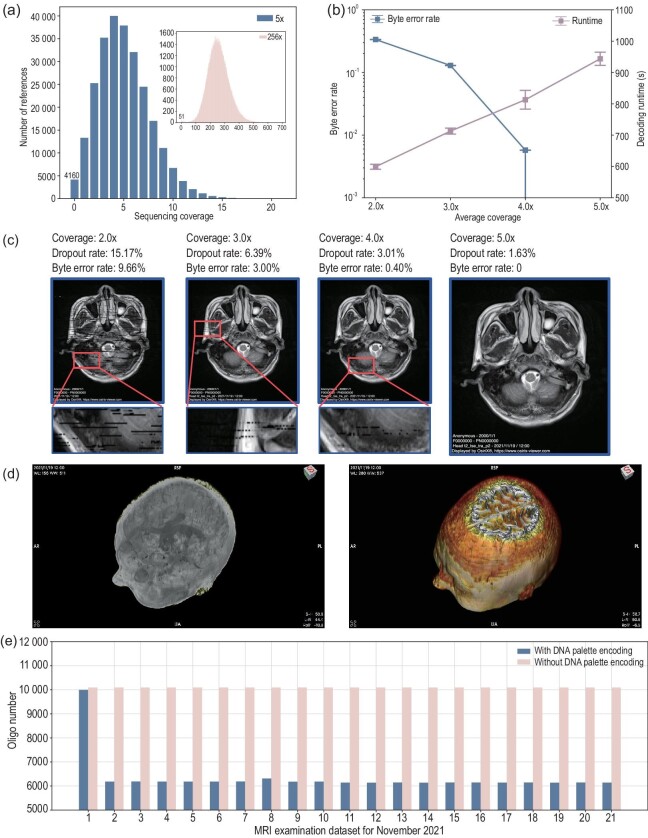
Experimental performance of our coding scheme. (a) Experimental results of the master pool with an average coverage of 256$\times$ and the dilution pool with an average coverage of 5$\times$. (b) Byte error rate and decoding time at average coverage ranges of 2$\times$, 3$\times$, 4$\times$ and 5$\times$. At each coverage rate, 10 independent experiments were conducted. (c) Reconstructed image pixel data using our method. (d) Maximum intensity projection image and three-dimensional volume rendering image reconstructed from the decoded data. (e) The number of oligos encoded from 21 DICOM files obtained from the November 2021 MRI examination, including results encoded by the DNA palette and indexed RS codes, is presented. The oligo count of the first file, which did not undergo differential encoding, showed a slight reduction. Other files exhibited a significant reduction in the number of encoded oligos.

In the *in vitro* experiment, we stored the medical imaging data from two brain MRI examinations of a patient with ischemic cerebrovascular disease conducted in November 2021 and October 2023. Each examination produced 21 DICOM files (Data S1). The 42 DICOM files, totaling 11.28 MB, were encoded into 255 248 oligos, each with a length of 155 nt (Fig. S3). The encoded oligos were synthesized by Twist Bioscience. The DNA pool was amplified through polymerase chain reaction, followed by a sequencing procedure on the Illumina sequencing platform. The mean coverage (i.e. the total number of reads divided by the number of encoded oligos) was 256 reads (Fig. 2a).

During the decoding process, we randomly sampled sequencing reads and gradually increased the sampling coverage. The sampling command is shown in Section S3.4. When the mean value of sampling coverage is 5$\times$, the dropout rate is 1.63$\%$ (Fig. 2a). As the average sequence coverage increases, the byte error rate (i.e. the byte error rate in the decoded output) decreases significantly, and the decoding time increases linearly (Fig. 2b). The minimum average coverage rates of the two examination files are 4.2$\times$ and 4.6$\times$ with a median of 4.4$\times$ (Table 1). The format of the elements is the number of successful decoding times compared to the number of tests. The specific experimental results are shown in Tables S1–S4. Images generated from decoded pixel information at different coverage rates are shown in Fig. 2c. In the presence of byte errors, mosaic artifacts may appear in the image; however, most of the information is still discernible. This shows that our decoder can recover part of the original data when only a partial sequencing read is received. It differs from compression algorithms such as DEFLATE, where even minor fragment loss can render the compressed data completely unrecoverable [4,26]. The decoded data can be used for three-dimensional (3D) reconstruction of medical images, such as the maximum intensity projection image and 3D volume rendering image (Fig. 2d). Besides, the number of encoded oligos for the first MRI examination is less than the index-added method (Fig. 2e). Here, ‘without DNA palette encoding’ denotes the scenario where the raw information is sequentially encoded into oligos, and the oligo index is added. The redundancy of its error-correcting code is the same as that of the DNA palette code. This suggests that, for MRI data, the DNA palette code can encode information using fewer oligos.

**Table 1.** Minimum coverage rates for decoding.

	Average coverage					
	4.2$\times$	4.4$\times$	4.6$\times$	4.8$\times$	5$\times$	Minimum average coverage
November 2021	5/10	8/10	8/10	10/10	10/10	4.2
October 2023	0/10	0/10	2/10	7/10	10/10	4.6

### Simulation on large data scales and diverse data formats

**Figure 3.**
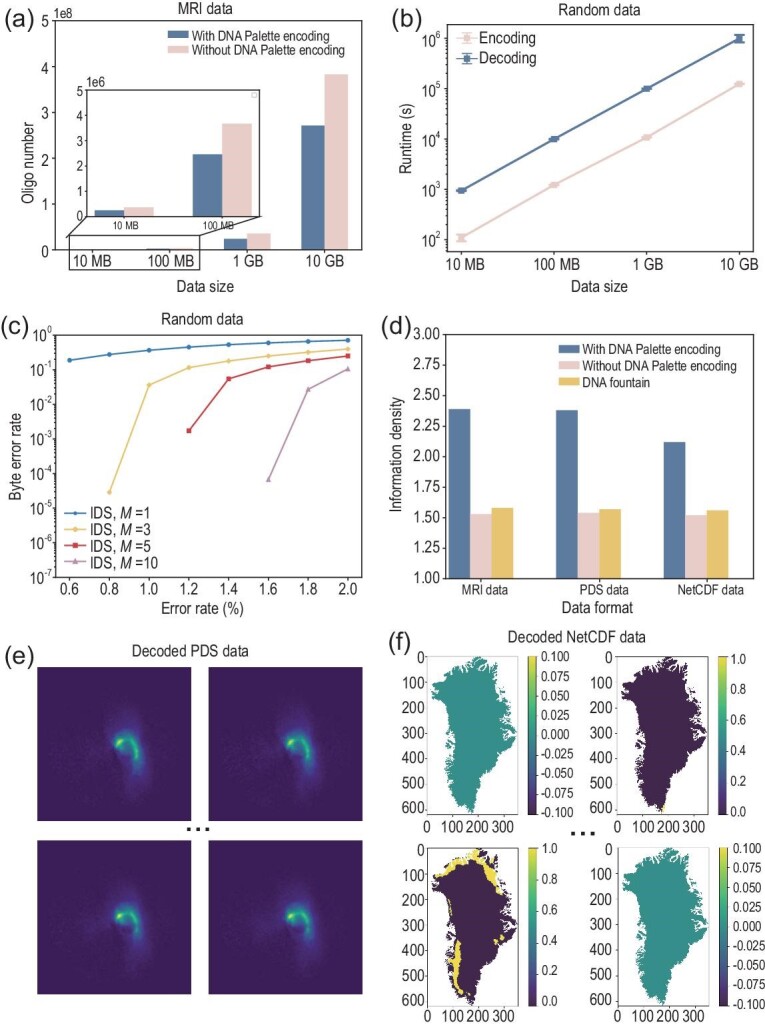
Simulations on large arge data scales and diverse data formats. (a) Number of encoded oligos (150 nt) in public brain MRI datasets with or without DNA palette encoding. (b) Encoding and decoding runtimes of our code on random data. Results are presented as mean values from 10 independent simulations. (c) Byte error rate of our method with IDS error rate ${\rm pr} = p_{\rm sub} + p_{\rm del} + p_{\rm ins}$, where $p_{\rm sub} : p_{\rm del} : p_{\rm ins} = 1 : 1 : 1$. Results are expressed as the average of 10 independent simulations (1-GB random data). The standard deviation values are too small to be clearly visualized. (d) Comparison of net information density among the encoding results of the DNA palette code, the same coding redundancy without the DNA palette code and the DNA fountain code in three data formats (MRI format, PDS format, NetCDF format). (e) Decoded pixel information of part of example PDS format files (Earth’s plasmasphere observed by the extreme ultraviolet camera on the Chang’E-3 Moon lander). (f) Decoded pixel information of part of example NetCDF format files (daily melt results for days 1, 100, 200 and 300 on the Greenland ice sheet surface in 1985).

First, to evaluate the performance of our coding scheme under a large data scale, we collected 10-GB DICOM files from public MRI datasets [27]. Simulations show that the DNA palette code has a stable effect on reducing the number of encoded oligos (Fig. 3a). When the coding redundancy is fixed, the number of encoded oligos can be reduced by approximately $1/3$ through DNA palette coding. We further conducted a series of simulations on random data with sizes ranging from 10 MB to 10 GB. The encoding time and the decoding time of the DNA palette code are linear in the length of the input (Fig. 3b). This is consistent with the results of the theoretical analysis. Our coding scheme also performs well in error handling across different DNA error rates and the number of duplicate sequencing reads (Fig. 3c). Here, we assumed that the IDS error rates are equal, and the total error rate is ${\rm pr}= p_{\rm ins}+p_{\rm del}+p_{\rm sub}$. The dropout rate $p_{\rm drop}=5\%$ and the duplication number *M* ranges in $\lbrace 1,3,5,10\rbrace$. We also tested the byte error rates when there were only substitution, dropout, deletion and insertion errors (Figs S9–S11). Experimental data analysis based on Twist synthesis and Illumina sequencing technology shows that the raw error rate of the DNA data storage system is less than $1\%$ [4,9,11]. Simulations indicate that our coding scheme can achieve error-free decoding at such an error rate.

We further tested our coding scheme on many different data formats. The first is the Planetary Data System (PDS) data format, which is a standardized form used for the archiving and distribution of planetary science data. Files in Table 2 record the Earth’s plasmasphere observations acquired by the extreme ultraviolet camera onboard the Chang’E-3 lander (Data S2) [28]. The second is the NetCDF Network Common Data Form (NetCDF), which is a software library and self-describing machine-independent data format that supports the creation, access and sharing of array-oriented scientific data. Files in Table 3 are based on the threshold method of the microwave radiometer’s day and winter brightness temperature difference to extract the Greenland ice sheet surface melt from the downscaling results, and obtain the $0.05^\circ$ daily melt results of the Greenland ice sheet surface in 1985, 2000 and 2015 (Data S3) [29–34]. Encoding results show that our coding scheme works well for such time-series archival datasets. Compared with the DNA fountain code, our code can effectively reduce the number of encoded nucleotides (Fig. 3d). Here, ‘$r({b}/{n})$’ refers to the ratio of the number of bits in the binary form of the file divided by the number of encoded nucleotides. Simulations show that our coding scheme is capable of error-free decoding in a wide range of data formats (panels (e) and (f) of Fig. 3).

**Table 2.** Encoding results for PDS files.

		PDS files										
		1	2	3	4	5	6	7	8	9	10	Total
Data size (KB)		49.2	49.2	49.2	49.2	49.2	49.2	49.2	49.2	49.2	49.2	492.2
$r({b}/{n})^{a}$												
With the DNA palette code		2.15	2.40	2.40	2.41	2.41	2.39	2.40	2.42	2.41	2.41	2.38
Without the DNA palette code		1.54	1.54	1.54	1.54	1.54	1.54	1.54	1.54	1.54	1.54	1.54

$^{a}$ Here $r({b}/{n})$ is the number of bits in the binary form of the file divided by the number of encoding nucleotides.

**Table 3.** Encoding results for NetCDF files.

		NetCDF files			
		1	2	3	Total
Data size (MB)		76.3	91.4	86.4	254.1
$r({b}/{n})$					
With the DNA palette code		2.19	2.11	2.10	2.12
Without the DNA palette code		1.52	1.52	1.52	1.52

## DISCUSSION

In the DNA data storage system, short DNA strands are stored in a spatially disordered structure within a three-dimensional space. Decoders typically require additional information to determine the order of the DNA strands to restore the original bitstream. The DNA palette code is designed to accommodate this feature. It is based on a sampling without replacement method, using unordered combinations of oligos to indicate binary information. The encoded oligos do not contain indexes and are not fixed in number when encoding different binary strings of the same length. When the DNA palette code is combined with contextual transformation methods (such as our proposed DNA ladder code), we can encode time-series archival data with fewer oligos than expected.

Additionally, rather than employing a compression algorithm, we developed a direct transcoding method that converts raw information bits to nucleotides. Our coding scheme demonstrates resilience in recovering information even in scenarios characterized by high dropout rates and byte error rates. Specifically, even when the received sequencing data are significantly insufficient and the error rate is high, such as when the dropout rate exceeds $15\%$ and the byte error rate exceeds 9% (Fig. 2c), our code can recover part of the raw information. In addition, our method can achieve a net information density of more than 2 bits/nt when encoding time-series archival data, which has the effect of a compression algorithm, while avoiding the problem of being unable to recover information when there are a small amount of residual errors in the decoded data due to the use of a compression algorithm (such as the DEFLATE algorithm).

We verified the effectiveness of our code in DNA data storage systems through wet and dry experiments. In our wet experiment, we encoded 11.28-MB MRI data into 255 248 oligos with a length of 155 nt, whereas the expected oligo number is 397 972. As shown in Table 4, compared with other studies, our wet experiment stored an average of 2.39 bits in one nucleotide and achieved 100$\%$ data recovery at a median decoding coverage of 4.4$\times$. In simulations, the reliability of our method has been verified on large data scales and diverse data formats.

**Table 4.** Comparison of DNA data storage experimental results and key achievements.

	Input data	Oligo	Number of		Average
Parameter	size (MB)$^{a}$	length$^{a}$	oligos$^{a}$	$r({b}/{n})$	coverage$^{a}$
Church *et al.* [1]	0.65	115	54 898	0.83	3000$\times$
Goldman *et al.* [2]	0.63	153	117	0.29	51$\times$
Erlich and Zielinski [11]	2.15	152	72 000	1.57	10.5$\times$
Organick *et al.* [4]	200.2	150–154	134 000 000	1.10	5$\times$
Ping *et al.* [26]	0.24	160	8087	1.56	1000$\times$
Song *et al.* [12]	6.8	164	210 000	1.58	N/A
**This work**	**11.28**	**155**	**255 248**	**2.39**	**4.4$\times$**

$^{a}$ The information presented is derived from the original data provided in the references. These references employed different original designs, including input files, parameter designs and experimental setups. This table is intended to provide a general comparative overview. ‘N/A’ indicates that the corresponding data are not available in the references.

The decoding results from wet experiments clearly reveal the presence of an acute cerebral infarction in the right frontal lobe of the patient in the medical images from November 2021 (Fig. S5). In the medical images from October 2023, this cerebral infarction has evolved into a liquefied lesion, and a new acute cerebral infarction has appeared in the left cerebellar hemisphere (Fig. S6). This highlights the significance of our scheme in storing medical images for disease screening and tracking.

The DNA palette code aligns with the spatial disorder of oligos, using unordered combinations of oligos as codewords. For binary input strings of the same length, the number of encoded oligos varies and does not require additional indexing. It performs well in both *in vitro* and *in silico* experiments and shows the potential to increase the information density and reduce decoding sequence coverage. The DNA palette code has the potential to expand new application scope for DNA data storage, offering robust support for historical trend analysis and retrospective studies. However, achieving the same high compression ratio as mature compression algorithms, such as the DEFLATE algorithm, presents a challenge for our encoder. This is an important direction for our next research.

## Supplementary Material

nwae321_Supplemental_Files

## Data Availability

The raw sequencing data are deposited in the figshare database under the following DOI link: https://doi.org/10.6084/m9.figshare.25131071. The Python implementation of the DNA palette code is available at https://github.com/ZihuiYan/DNA-Palette-code.git.
