# Supplementary material for: DNA palette code for time-series archival data storage
Source: Natl Sci Rev. 2024 Sep 10;12(1):nwae321. doi: 10.1093/nsr/nwae321 (PMC11697981; doi:10.1093/nsr/nwae321)
Supplement: nwae321_Supplemental_Files [file nwae321_supplemental_files.zip › Supplementary Materials.pdf]

Supplementary Materials for

DNA Palette Code for Time-Series Archival Data Storage

Zihui Yan[1,2†], Haoran Zhang[1,2†], Boyuan Lu[1,2], Tong Han[3], Xiaoguang Tong[3*], Yingjin Yuan[1,2*]

[1]Frontiers Science Center for Synthetic Biology and Key Laboratory of Systems Bioengineering (Ministry of Education) Tianjin University, Tianjin, 300072, China.
[2]School of Chemical Engineering and Technology, Tianjin University, Tianjin, 300072, China.
[3]Department of Neurosurgery, Huanhu Hospital, Tianjin, 300350, China.

*Corresponding author(s). E-mail(s): xg_tong@139.com; yjyuan@tju.edu.cn;
†These authors contributed equally to this work.

This PDF file includes:

Methods and Materials

References

Figs. S1 to S12

Tables S1 to S5

Caption for Data S1 to S3

Caption for Movies S1 to S3

Other Supplementary Material for this manuscript includes the following:

Data S1, Data S2, Data S3

Movie S1, Movie S2, Movie S3

Materials and Methods

1 Details of the DNA Palette coding scheme

1.1 Nested coding scheme

Given the challenges associated with storing and manipulating a large mapping alphabet, we introduced a solution called the nested mapping. When mixing colors for oil painting, the resulting color is influenced not only by the primary colors used but also by the quantity and texture of those colors. Inspired by this, we developed a method to combine oligos across multiple dimensions to represent more diverse information. Firstly, we defined two preset oligo sets $O_1 = \{\boldsymbol{o^1_1}, \boldsymbol{o^1_2}, \ldots, \boldsymbol{o^1_n}\}$ and $O_2 = \{\boldsymbol{o^2_1}, \boldsymbol{o^2_2}, \ldots, \boldsymbol{o^2_k}\}$, representing primary colors and available textures, respectively. We assumed that each color is allowed to be used no more than t times. Specifically, the set of mixing colors is $O_1 \circ O_2 = \{\boldsymbol{o^1_{i_1}} \circ \boldsymbol{o^2_{i_2}} \mid \boldsymbol{o^1_{i_1}} \in O_1, \boldsymbol{o^2_{i_2}} \in O_2\}$, where $\boldsymbol{o^1_{i_1}} \circ \boldsymbol{o^2_{i_2}}$ indicates a mixed color that combines the color $\boldsymbol{o^1_{i_1}}$ with the texture $\boldsymbol{o^2_{i_2}}$, formed by splicing $\boldsymbol{o^1_{i_1}}$ and $\boldsymbol{o^2_{i_2}}$. Given this method of generating mixed colors through random and non-repeating combinations of oligos, these colors can then be used to encode distinct binary information.

The number of mixing colors is given by $\left(\sum_{j=0}^{t} \binom{k}{j}\right)^n$, and the minimum length of oligos in $O_1 \circ O_2$ is $(\lceil \log_4 n \rceil + \lceil \log_4 t \rceil)$. Let $M(\boldsymbol{x})$ denote the number of primary colors used in a mixing color. For any oligo in O_1, it can be selected between 0 and t times, ensuring that $0 \leq M(\boldsymbol{x}) \leq nt$.

Given the method of creating mixing colors—using unordered and non-repeating combinations of oligos—we can represent different binary information by establishing a one-to-one mapping between oligo sets and binary data. Specifically, for any $A = O_1^A \circ O_2^A \subset O_1 \circ O_2$ and $B = O_1^B \circ O_2^B \subset O_1 \circ O_2$, the order $<$ is defined as follows: (i) if $O_1^A < O_1^B$, then $A < B$; (ii) if $O_1^A = O_1^B$ and $O_2^A < O_2^B$, then $A < B$; (iii) otherwise, $B < A$. According to this order, $\mathcal{O}_1 \circ \mathcal{O}_2$ (i.e., the set family of $O_1 \circ O_2$) forms a totally ordered set. We define a map $g : \mathcal{X}^N \to \mathcal{O}_1 \circ \mathcal{O}_2$ such that $g(\boldsymbol{x})$ is the $D(\boldsymbol{x})$-th oligo set in $\mathcal{O}_1 \circ \mathcal{O}_2$, where $D(\boldsymbol{x})$ represents the decimal form of $\boldsymbol{x}$. Furthermore, when the size of the range of g is restricted to 2^N (i.e., the first 2^N sets in $\mathcal{O}_1 \circ \mathcal{O}_2$), g is reversible, making g a non-singular coding mapping.

The code rate of the above coding mapping is

$$R_g = \frac{n \log_2 \left(\sum_{j=0}^{t} \binom{k}{j} \right)}{\mathbb{E}\left(M(\boldsymbol{x})\right) \left(\lceil \log_4 n \rceil + \lceil \log_4 k \rceil \right)}. \tag{1}$$

The preset oligos in O_1 and O_2 can be designed according to user requirements. In our experiment, the encoded oligo set $O_1 \circ O_2$ is generated through the following steps: (i) We selected $\mathcal{D}^{\lceil \log_4 n \rceil}$ and $\mathcal{D}^{\lceil \log_4 t \rceil}$ as the initial sets. The set $O_1 \circ O_2$ is defined as the set of oligos in O_2 periodically inserted into oligos in O_1. (ii) Each sequence is then combined with a predetermined pseudo-random sequence using a bit-wise XOR operation. This pseudo-random sequence can be user-defined or generated by a pseudo-random number generator using the Mersenne Twister algorithm [1]. (iii). To combat IDS errors, the sequences in $O_1 \circ O_2$ are designed to conform to the VT structure [2–4]. The definition of the VT structure is elucidated in Section 1.2.

1.2 VT structure in the DNA Palette code

The typical binary VT code is a single IDS error correction code. It can be described as a class of binary algebraic block codes that consists of all binary strings of length n belonging to

$$VT_{a,2n+1}(n) = \left\{ \boldsymbol{x} \in \{0,1\}^n : \sum_{i=1}^{n} i x_i \equiv a \pmod{2n+1} \right\}, \tag{2}$$

where a is an integer with $0 \le a \le 2n$, usually called the syndrome. Levenshtein [5] found that this code is an asymptotically optimal single-IDS error correction code. In this work, we introduced a generalized form of the VT code, named the interleaved quaternary VT code. For a quaternary symbol x_i, let $x_i = x_i^{(0)} 2^0 + x_i^{(1)} 2^1$ be the binary expansion for symbol x_i. Then a quaternary sequence $\boldsymbol{x} = (x_1, x_2, \cdots, x_n) \in \Sigma^n$ can be expanded as two binary sequences $\boldsymbol{x^{(0)}} = (x_1^{(0)}, \cdots, x_n^{(0)})$ and $\boldsymbol{x^{(1)}} = (x_1^{(1)}, \cdots, x_n^{(1)})$. We define $\boldsymbol{x}$ as the interleaved sequence of $\boldsymbol{x^{(0)}}$ and $\boldsymbol{x^{(1)}}$, denoted by $\boldsymbol{x^{(1)}} \parallel \boldsymbol{x^{(0)}}$. Then the quaternary VT structure can be generated by interleaving two binary VT codewords. We introduced the systematic encoder of the binary VT code designed by Saowapa *et al.* [6]. To be specific, for a binary sequence $\boldsymbol{x} = \{x_1, x_2, \cdots, x_k\} \in \{0,1\}^k$, the VT encoder inserts it into a codeword $\boldsymbol{y} \in VT_{a,2n+1}(n)$, where $k = n - \lceil \log n \rceil - 1$. The encoder inserts "parity" bits at dyadic positions, i.e., c_{2^i}, for $0 \le i \le t-2$ and c_n, and attaches message symbols to

other positions, to ensure that $\sum_{i=1}^{n} iy_i \equiv a \pmod{2n+1}$. Here, $t = n - k$ is the number of redundancy bits.

Example: For a binary sequence $\boldsymbol{x} = 01010$ and a fixed syndrome $a = 0$, it follows $n = 10$, and $t = 5$. The codeword $\boldsymbol{y} = (y_1, y_2, \cdots, y_{10})$ should satisfy that $\sum_{i=1}^{10} iy_i \equiv \sum_{j=1}^{4} 2^{j-1} y_{2^{j-1}} + 10 \cdot y_{10} + 0 \cdot 3 + 1 \cdot 5 + 0 \cdot 6 + 1 \cdot 7 + 0 \cdot 9 \equiv 0 \pmod{21}$. Then expand $21 - 12 = 9$ into the binary form $1 \cdot 2^3 + 1 \cdot 2^0$. We obtain the codeword $\boldsymbol{y} = \bar{1}\bar{0}0\bar{0}101\bar{1}0\bar{0}$, where bits with overbars are check bits.

The quaternary VT structure is defined as follows,

$$\text{Q-VT}_{k,a}(n) = \left\{ \boldsymbol{x} = \boldsymbol{x}^{(1)} \parallel \boldsymbol{x}^{(0)} : \boldsymbol{x}^{(i)} \in VT_{a,2n+1}(n), i = 0, 1 \right\}, \tag{3}$$

where $k = n - \lceil \log_2 n \rceil - 1$. The aforementioned single-IDS error correction code operates over the quaternary alphabet. Analysis of experimental data from the Twist synthesis process and the Illumina sequencing process revealed that the DNA data storage system exhibited a low raw error rate of nearly 1% [7–9]. The length of the oligos is typically less than 300 nt due to synthesis technology limitations. Overall, the error-correcting capability of VT codes, combined with duplicate sequencing reads, is effective in handling such error scenarios. Moreover, the VT codes have asymptotically optimal coding rates and linear encoding and decoding complexity. This makes VT codes well-adapted to DNA data storage.

1.3 Data pre-processing method: the DNA Ladder code

DICOM is a standard for the digital transmission, display, and storage of medical images developed in 1985 by American College of Radiology and National Electrical Manufacturers Association. DICOM files typically contain protected health information (PHI) about a patient, such as name, gender, age, and other image-related information, such as details of the device used to capture and generate the image and context-specific medical information (Fig. S3). By examining the unique format of DICOM data, we observed that the PHI segment in a set of DICOM files tends to exhibit significant similarities. This inspires us to first align the labels of the DICOM file, and then use differential encoding to update the data. Specifically, during the differential encoding process, the binary representation of the first file remains unchanged, and the binary representation of each subsequent file performs a bitwise XOR operation with the previous sequence.

This will produce a new binary string with a lower Hamming weight. Furthermore, recognizing that error-prone processes such as DNA synthesis, preservation, amplification, and sequencing are inevitable, we introduced RS codes to embed parity-check information into binary strings. To sum up, DNA Ladder encoding includes three steps: label alignment, differential encoding, and RS encoding.

The (n, k) RS code is one of the widely studied erasure codes. Theoretically, it can recover fewer than $n - k$ erasure errors or correct fewer than $(n - k)/2$ substitution errors. However, the codewords generated by the DNA Palette code lack indexes, making it impossible to determine the location of dropout errors. Consequently, dropout errors cannot be converted into erasure errors, leading the RS code to treat all errors as substitution errors. In our coding scheme, we incorporated the $(255, 223)$ RS code to encode the binary string blocks following the differential encoding process. It can correct up to 6.27% of substitution errors.

After combining DNA Ladder code and DNA Palette code, the code rate of our encoding scheme is

$$R_{Ladder-Palatte} = \frac{223}{255} \cdot \frac{n \log_2 \left(\sum_{j=0}^{t} \binom{k}{j} \right)}{\mathbb{E}\left(M(\boldsymbol{x})\right) \left(\lceil \log_2 \left(\lceil \log_4 n \rceil + \lceil \log_4 k \rceil \right) \rceil + \lceil \log_4 n \rceil + \lceil \log_4 k \rceil + 1 \right)}. \tag{4}$$

In our in vitro experiments, we selected $k = 2^{272}$ and $t = 1$, resulting in the code rate of 1.54 bits per nucleotide.

However, for a special binary string $\boldsymbol{x}$ that satisfies $M(\boldsymbol{x}) < nt$, the actual number of encoded oligos decreases. Then, we can use one nucleotide to store $\frac{223n}{255M(\boldsymbol{x})} \cdot \frac{\log_2(k+1)}{(\lceil \log_2(\lceil \log_4 n \rceil + \lceil \log_4 k \rceil) \rceil + \lceil \log_4 n \rceil + \lceil \log_4 k \rceil + 1)}$ bits. The practical information density exceeds 1.54 bits/nt because $\frac{M(\boldsymbol{x})}{n} \approx \frac{2}{3}$ when encoding the MRI dataset we collected (Fig. 3a).

1.4 Testing on more data formats

We have tested the practicality of our encoding scheme on MRI files, PDS files, and NetCDF files. However, there are other data types that are time-series, such as surveillance camera video files. We encoded 20 frame-by-frame surveillance images (.bmp) in the Segmenting and Tracking Every Pixel (STEP) benchmark files [10]. Each encoded nucleotide stored 2.12 bits (Table S5).

However, the requirements for data recovery accuracy and clarity in video files from surveillance cameras are lower than those for scientific data. To optimize storage density for these applications, future work should focus on developing specialized data preprocessing methods, including effective lossy compression techniques. That will enable us to achieve higher density storage tailored to the specific needs of different types of data.

Additionally, our encoding algorithm is applicable to compressed files. We compressed the DICOM files used in our in vitro experiment using the DEFLATE algorithm. The ratio of the number of bits in the compressed files to the number of nucleotides encoded by the Palette code is 1.538, representing an improvement over the encoding method that does not utilize the Palette scheme (i.e., 1.537). However, due to the characteristics of the compression algorithm, the decoder is very sensitive to residual byte errors.

2 Method

2.1 Reagents

Oligo pools were synthesized by Twist Bioscience. Primers were ordered from Genewiz (Azenta Life Sciences). The KAPA HiFi HotStart PCR Kit (Cat#KK2502) was sourced from Roche. UltraPure water (Cat#10977015) and UltraPure 1M Tris-HCl, pH 8.0 (Cat#15568025), were purchased from Thermo Fisher Scientific. The 10 mM Tris Buffer (pH 8.0) was diluted from 1M Tris-HCl using UltraPure water. SPRI magnetic beads (Cat#B23318) were ordered from Beckman Coulter Life Sciences.

2.2 PCR amplification and NGS-sequencing of oligo pools

The oligo pool was carefully and thoroughly dissolved in 10 mM Tris Buffer (pH 8.0), achieving a final concentration of 10 ng/µL. The following PCR components were accurately blended: 1 µL of the oligo pool (10 ng/µL), 1.5 µL of KAPA dNTP Mix (10 mM each), 1.5 µL of 10 µM forward primers (with overhang of indexed P5 Illumina adapters), 1.5 µL of 10 µM reverse primers (with overhang of indexed P7 Illumina adapters), 10 µL of 5X KAPA HiFi Fidelity Buffer, 1 µL of KAPA HiFi HotStart DNA Polymerase (1 U/µL), and 33.5 µL of ultrapure water to reach a total volume of 50 µL. The PCR amplification procedure included an initial denaturation at 95°C for 3 minutes, followed by 12 cycles of 98°C for 20 seconds, 60°C for 20 seconds, and 72°C for 15 seconds, concluding with a final extension at 72°C for 1 minute. PCR products were cleansed using SPRI®

magnetic beads at a 1.8x bead-to-DNA ratio. Subsequently, the Qsep400 Bio-Fragment Analyzer was employed to determine the length of the purified PCR products, confirming a clean peak at the expected size (Fig. S12). Indexed Illumina adapters (P5 and P7) were attached via PCR using overhanging forward and reverse primers, allowing the PCR products to be directly utilized for NGS sequencing. The 150-cycle paired-end sequencing using the Illumina NovaSeq 6000 platform yielded about 10 Gb of data, averaging 241 paired reads per sequence.

Forward primer sequence for oligo pool 1:

5'-AATGATACGGCGACCACCGAGATCTACACACCTAGTCCTACACTCTTT CCCTACACGACGCTCTTCCGATCTCCACGCGTACCGATAGCTTCAG-3'

Reverse primer sequence for oligo pool 1:

5'- CAAGCAGAAGACGGCATACGAGATGGTAGATCGAGTGACTGGAGTT CAGACGTGTGCTCTTCCGATCTGATACATGCGTGGGTCAATTGC-3'

Forward primer sequence for oligo pool 2:

5'-AATGATACGGCGACCACCGAGATCTACACTTATCCGACGACACTCTTT CCCTACACGACGCTCTTCCGATCTCCACGCGTACCGATAGCTTCAG-3'

Reverse primer sequence for oligo pool 2:

5'-CAAGCAGAAGACGGCATACGAGATGAGGCGATAAGTGACTGGAGTT CAGACGTGTGCTCTTCCGATCTGATACATGCGTGGGTCAATTGC-3'

3 DNA Patelle code experiments

3.1 Experiments

All encoding and decoding experiments were done using a MacBook Pro with a 2.4 GHz Intel Core i9 and 64 Gbyte of memory. The code was tested with Python 3.8.5. Large-scale simulation is performed in a distributed manner. Error simulation involves randomly extracting sequences from the encoding results and inserting errors. The decoding process consists of three stages: 1. Distributing error sequences to decoding nodes and performing initial decoding; 2. Decoding nodes identify the file index, and mix all data belonging to the same file index; 3. Distributing data with the determined index to decoding nodes to recover files.

3.2 Installation

Code, data, and input files are provided online. The sequencing data generated in this study have been deposited in the figshare database under the following DOI links: https://doi.org/10.6084/m9.figshare.25131071. A Python implementation of the DNA Palette code is available at https://github.com/ZihuiYan/DNA-Palette-code.git.

3.3 Command line steps to encode and decode

The step-by-step commands:

```
# Encoding for DICOM:
  python DICOM_encoder.py
  # Input is the folder of DICOM files. Output is a txt file, in which
each line is an oligo, named codeword.txt. It consists of 255,248 oligos
of 199nt, which included the 155nt payload flanked by landing sites for
sequencing primers:
            CCACGCGTACCGATAGCTTCAG[N155]GCAATTGACCCACGCATGTATC

# Decoding for DICOM:
```

One need to first download a partially sampled dataset of sequencing reads (https://doi.org/10.6084/m9.figshare.25567545.v1) and run the demo.

```
  python DICOM_decoder.py
  # Input is a txt file, in which each line is a sequencing read. Output
is DICOM files, saved in the folder named dec_files.

# Testing for random data:
  python example_test.py

# Testing parallel processes with random data:
  python setup.py build_ext --inplace
  python P_enc.py
  python P_error_mutation.py
  python P_dec_process_num.py
  python P_dec_Pelette.py
```

3.4 Raw read processing

Processing sequencing data, including filtering low-quality reads and trimming primers using fastp [11]:

```
fastp -i AP1_1.fq.gz -I AP1_2.fq.gz -o clean_AP1_1.fq.gz -O clean_AP1_2.fq.gz -h -q 20 -u 60 -A -c -g -f 22 -F 22
fastp -i AP2_1.fq.gz -I AP2_2.fq.gz -o clean_AP2_1.fq.gz -O clean_AP2_2.fq.gz -h -q 20 -u 60 -A -c -g -f 22 -F 22
```

Stitching reads using Flash [12]:

```
flash -m 98 -M 105 -x 0.01 -o flash_seq -d output clean_AP1_1.fq.gz clean_AP1_2.fq.gz
```

The length distribution of the sequences after paired-end assembly is shown in the Fig. S8.

```
flash -m 98 -M 105 -x 0.01 -o flash_seq -d output clean_AP2_1.fq.gz clean_AP2_2.fq.gz
```

Sequence filtering with bioawk:

```
bioawk -c fastx ’{if (length($seq) >= 154 && length($seq) <= 156) print "@"$name"\n"$seq"\n+\n"$qual}’ .\output\flash_seq.extendedFrags.fastq > clean_forward_1.fastq
bioawk -c fastx ’{if (length($seq) >= 154 && length($seq) <= 156) print "@"$name"\n"$seq"\n+\n"$qual}’ .\output\flash_seq.extendedFrags.fastq > clean_forward_2.fastq
```

Merging data from both sequencing pools:

```
cat clean_forward_1.fastq clean_forward_2.fastq > merged.fastq
```

Randomly sampling sequencing reads using seqtk:

```
seqtk sample -s 1000 merged.fastq 0.015 > subset_0.015.fastq
awk ’NR%4==2 {print}’ subset_0.015.fastq > sequences_0.015.txt
```

References

[1] Matsumoto, M., Nishimura, T.: Mersenne twister: a 623-dimensionally equidistributed uniform pseudo-random number generator. ACM Trans. Model. Comput. Simul. **8**, 3–30 (1998)

[2] Varšamov, R., Tenengolts, G.: A code which corrects single asymmetric errors. Annals of Telecommunications **26**(4), 288–292 (1965)

[3] Yan, Z., Liang, C., Wu, H.: A segmented-edit error-correcting code with re-synchronization function for DNA-based storage systems. IEEE Transactions on Emerging Topics in Computing (2022)

[4] Yan, Z., Qu, G., Wu, H.: A novel soft-in soft-out decoding algorithm for VT codes on multiple received DNA strands. In: 2023 IEEE International Symposium on Information Theory (ISIT), pp. 838–843 (2023)

[5] Levenshtein, V.I.: Binary codes capable of correcting deletions, insertions, and reversals. Soviet physics. Doklady **10**, 707–710 (1965)

[6] Saowapa, K., Kaneko, H., Fujiwara, E.: Systematic deletion/insertion error correcting codes with random error correction capability. In: Proceedings of the 14th International Symposium on Defect and Fault-Tolerance in VLSI Systems, pp. 284–292 (1999)

[7] Erlich, Y., Zielinski, D.: DNA fountain enables a robust and efficient storage architecture. Science **355**(6328), 950–954 (2017)

[8] Organick, L., Ang, S.D., Chen, Y.-J., Lopez, R., Yekhanin, S., Makarychev, K., Racz, M.Z., Kamath, G., Gopalan, P., Nguyen, B., *et al.*: Random access in large-scale DNA data storage. Nature Biotechnology **36**(3), 242–248 (2018)

[9] Heckel, R., Mikutis, G., Grass, R.N.: A characterization of the DNA data storage channel. Scientific Reports **9**(1), 1–12 (2019)

[10] Weber, M., Xie, J., Collins, M., Zhu, Y., Voigtlaender, P., Adam, H., Green, B., Geiger, A., Leibe, B., Cremers, D., Ošep, A., Leal-Taixé, L., Chen, L.-C.: STEP: Segmenting and Tracking Every Pixel (2021)

[11] Chen, S.: Ultrafast one-pass FASTQ data preprocessing, quality control, and deduplication using fastp. iMeta **2** (2023)

[12] Magoc, T., Salzberg, S.: FLASH: Fast length adjustment of short reads to improve genome assemblies. Bioinformatics (Oxford, England) **27**, 2957–63 (2011)

Supplementary figures

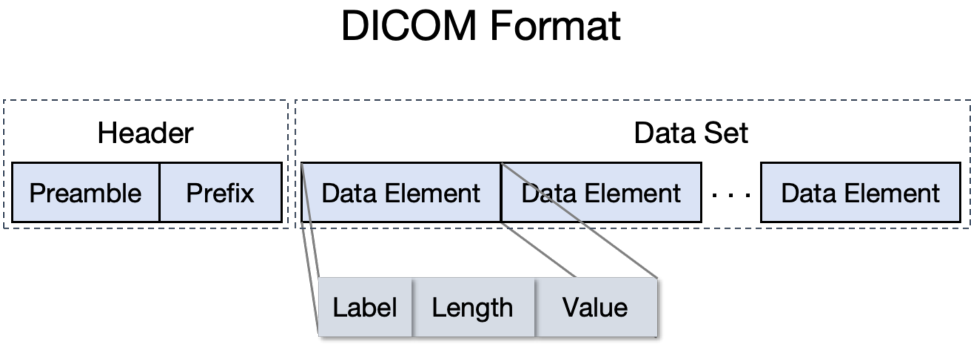

Fig. S1: Data format of DICOM files.

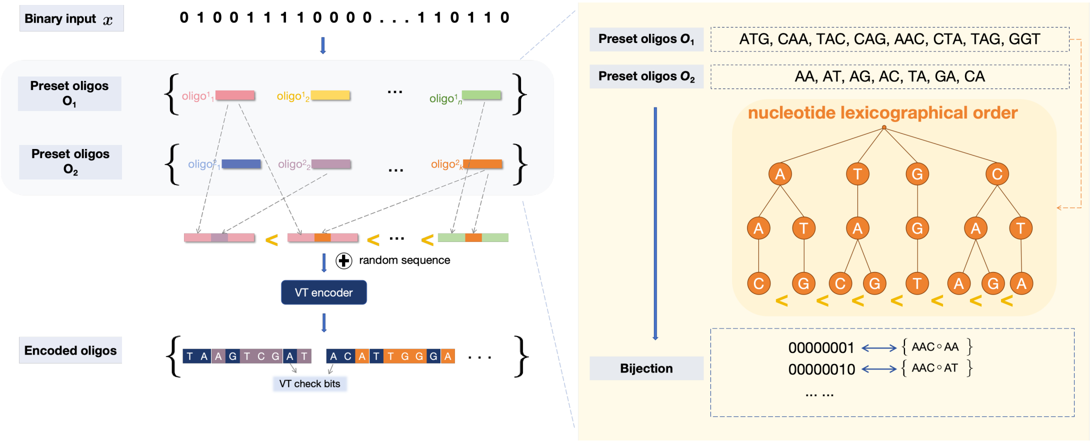

Fig. S2: The schematic diagram of the nested mapping.

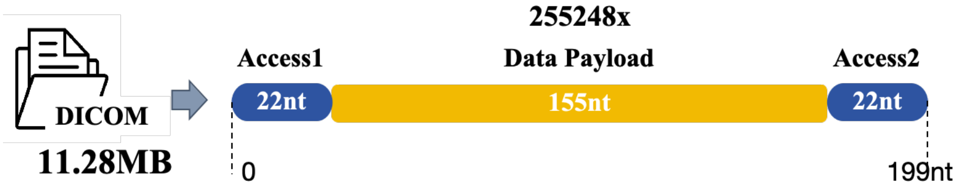

Fig. S3: Structure of the oligos. Access 1 and access 2 are landing sites for primers in the PCR amplification procedure.

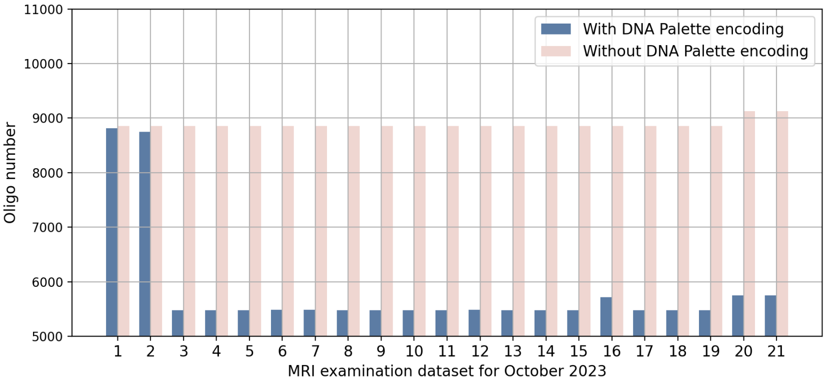

Fig. S4: Encoded oligo number of the October 2023 MRI scan.

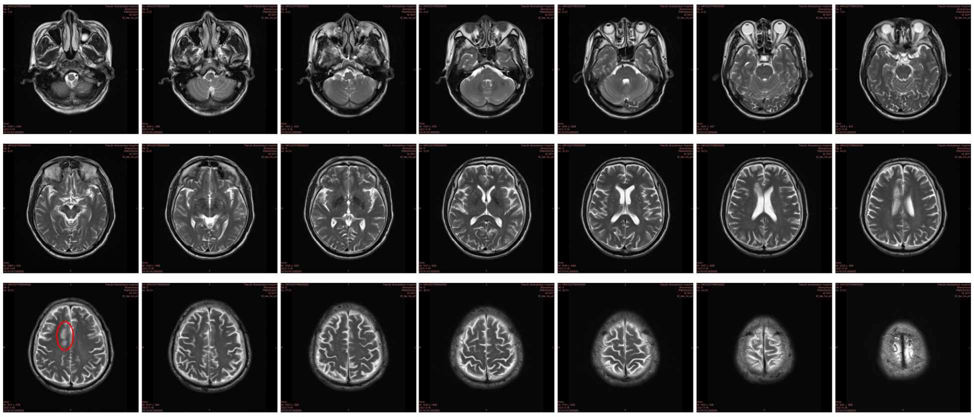

Fig. S5: Decoded images of the November 2021 MRI scan. The highlighted area in red indicates visible acute cerebral infarction lesions.

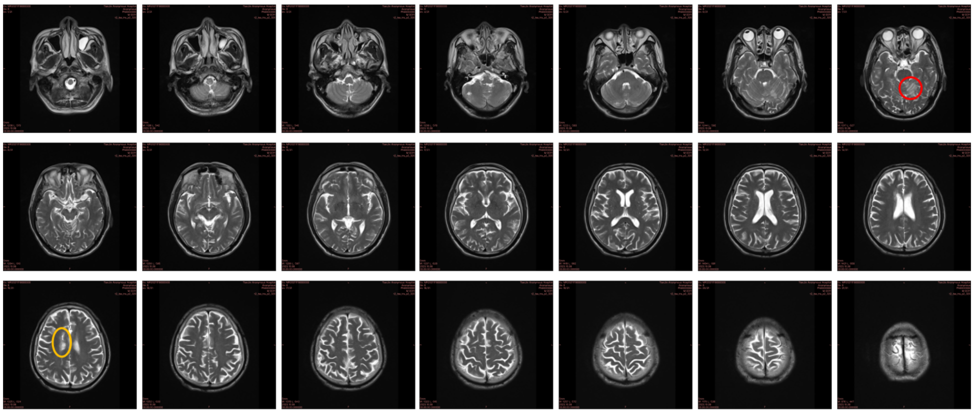

Fig. S6: Decoded images of the October 2023 MRI scan. The newly highlighted red portion reveals fresh acute cerebral infarction lesions, while the yellow-highlighted area indicates that the initial lesion has evolved into a malacia lesion.

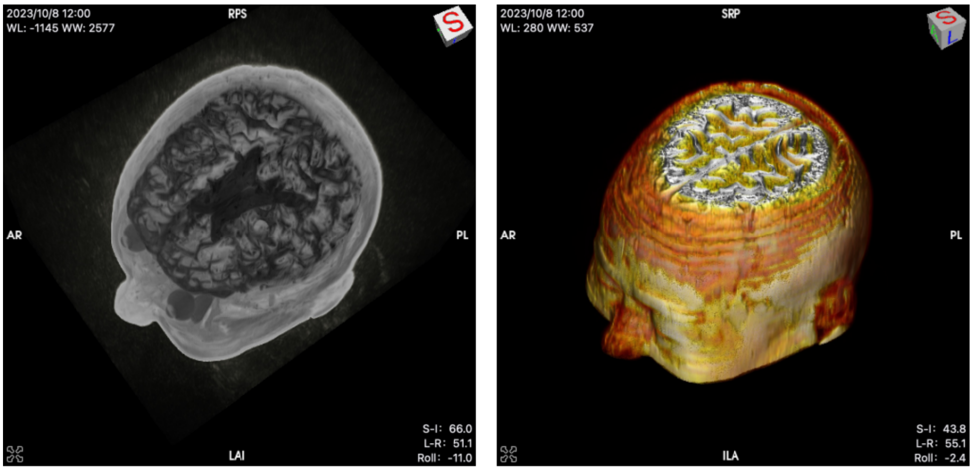

Fig. S7: Maximum intensity projection image and 3D volume rendered image reconstructed from decoded data of the October 2023 MRI scan.

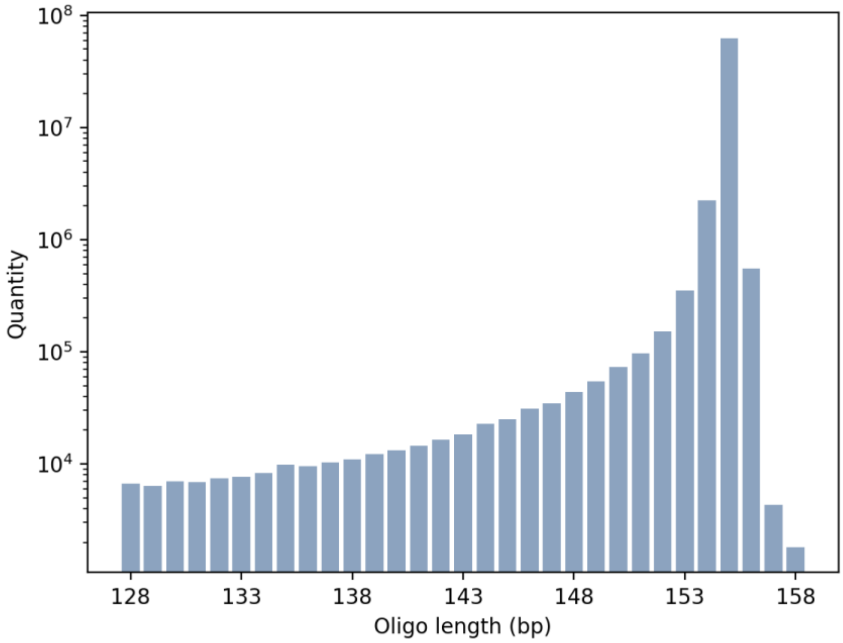

Fig. S8: BioAnalyzer results for oligo pools. The mode of the spliced fragment length is 155nt, which is consistent with the expected result.

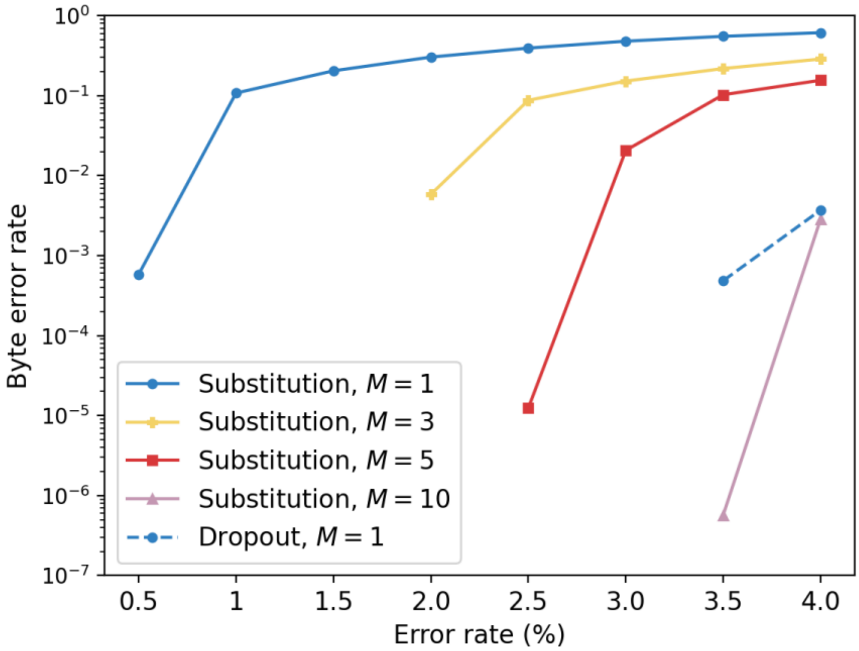

Fig. S9: Byte error rate of our code with only substitution errors/dropout errors. The results are presented as mean values from ten independent simulations. The standard deviation (SD) values are too small to be clearly visualized.

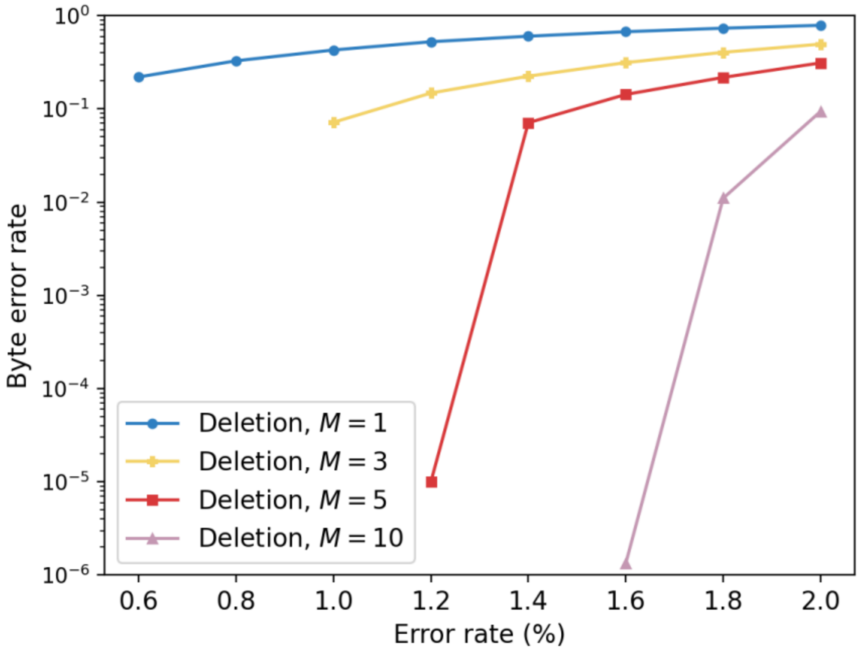

Fig. S10: Byte error rate of our code with only deletion errors. The results are presented as mean values from ten independent simulations. The standard deviation (SD) values are too small to be clearly visualized.

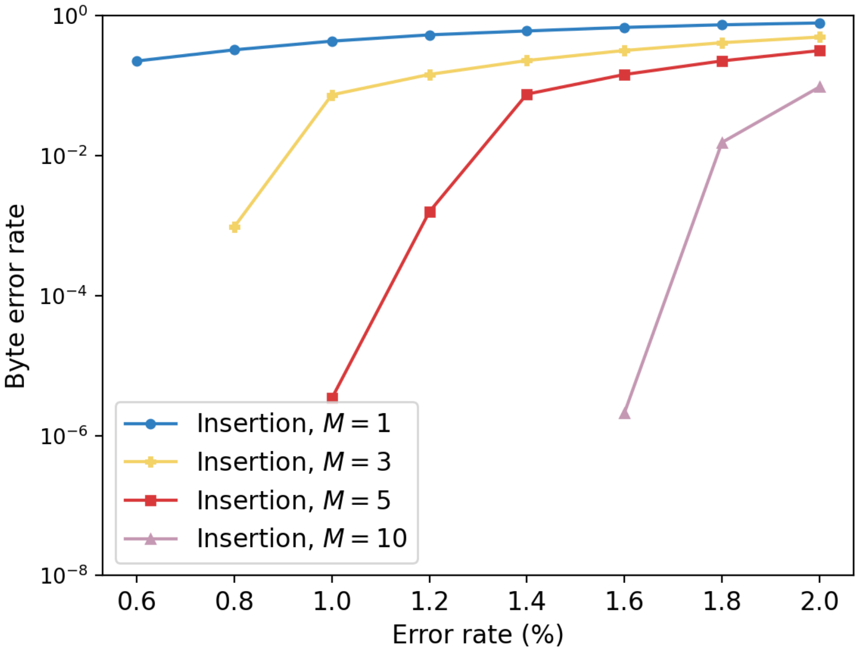

Fig. S11: Byte error rate of our code with only insertion errors. The results are presented as mean values from ten independent simulations. The standard deviation (SD) values are too small to be clearly visualized.

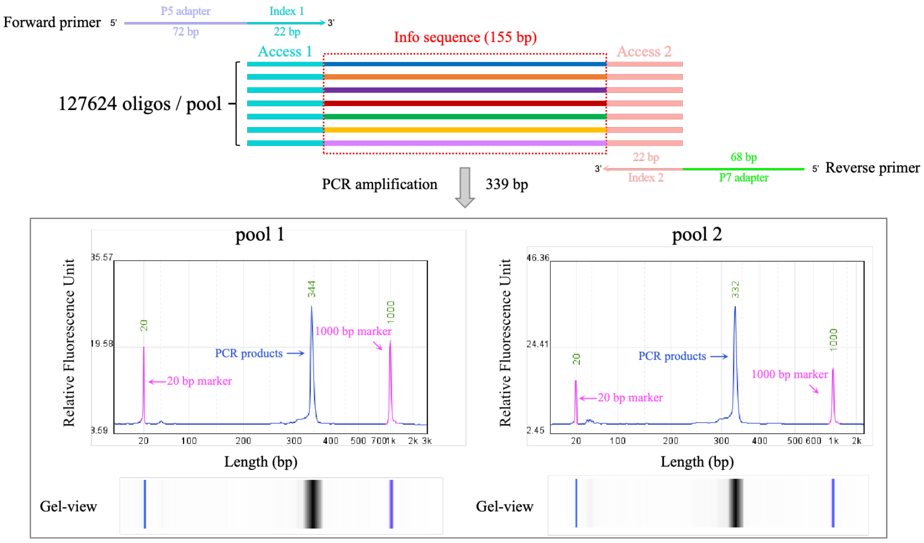

Fig. S12: The singular peak observed in the electropherogram through capillary electrophoresis signifies the presence of a distinct and pure PCR product measuring 339 bp in length.

Supplementary tables

Table S1: Byte error rate at average coverage of 4.2x.

		1	2	3	4	5	6	7	8	9	10
Sequencing reads number		1082015	1081881	1081761	1080634	1079960	1081607	1083366	1082128	1080604	1081192
Mean coverage		4.239073	4.238548	4.238078	4.233663	4.231022	4.237475	4.244366	4.239516	4.233545	4.235849
	0-th file	0	0	0.002506	0.001755	0	0	0	0	0.0033	0
	1-th file	0	0	0.002506	0.001755	0	0	0	0	0	0
	2-th file	0	0	0.002506	0.001755	0	0	0	0	0	0
	3-th file	0	0	0.002506	0.001755	0	0	0	0	0	0
	4-th file	0	0	0.002506	0.001755	0	0	0	0	0	0
	5-th file	0	0	0.002506	0.001755	0	0	0	0	0	0
	6-th file	0	0	0.002506	0.001755	0	0	0	0	0	0
	7-th file	0	0	0.002506	0.001755	0	0	0	0	0	0
	8-th file	0	0	0.002506	0.001755	0	0	0	0	0	0
	9-th file	0	0	0.002506	0.001755	0	0	0	0	0	0
November 2021	10-th file	0	0	0.002506	0.001755	0	0	0	0	0	0
	11-th file	0	0	0.002506	0.001755	0	0	0	0	0	0
	12-th file	0	0.00013	0.002506	0.001755	0	0	0	0	0	0
	13-th file	0	0.00013	0.002506	0.001755	0	0	0	0	0	0
	14-th file	0	0.00013	0.002506	0.001755	0	0	0	0	0	0
	15-th file	0	0.00013	0.002506	0.001755	0	0	0	0	0	0
	16-th file	0	0.00013	0.002506	0.001755	0	0	0	0	0	0
	17-th file	0	0.00013	0.002506	0.001755	0	0	0	0	0	0
	18-th file	0	0.00013	0.002506	0.001755	0	0	0	0	0	0
	19-th file	0	0.00013	0.002506	0.001755	0	0	0	0	0	0
	20-th file	0	0.00013	0.002506	0.001755	0	0	0	0	0	0.00035
	21-th file	0.003034	0	0.002197	0.00698	0.002327	0.001939	0.00181	0	0.00168	0
	22-th file	0.003034	0.002061	0.002197	0.009888	0.005782	0.003505	0.004254	0.001874	0.003791	0.002988
	23-th file	0.003034	0.002061	0.002197	0.009888	0.005782	0.003505	0.004254	0.001874	0.003791	0.002988
	24-th file	0.003034	0.002061	0.002197	0.009888	0.005782	0.003505	0.004254	0.001874	0.003791	0.002988
	25-th file	0.003034	0.002802	0.002197	0.010858	0.005782	0.003505	0.004254	0.001874	0.003791	0.002988
	26-th file	0.003927	0.003475	0.002247	0.010857	0.005782	0.003505	0.004254	0.001874	0.003791	0.002988
	27-th file	0.003927	0.004174	0.002247	0.010857	0.005782	0.004588	0.004254	0.001874	0.004753	0.002988
	28-th file	0.005083	0.004174	0.002368	0.011751	0.006125	0.004588	0.004254	0.001874	0.004753	0.002988
	29-th file	0.005083	0.004174	0.003326	0.011751	0.007269	0.004588	0.004254	0.001874	0.004753	0.002988
	30-th file	0.005083	0.004174	0.003326	0.011751	0.007269	0.004588	0.004254	0.001874	0.004753	0.002988
October 2023	31-th file	0.005083	0.004174	0.003326	0.011751	0.007269	0.004588	0.004254	0.001874	0.004753	0.002988
	32-th file	0.006159	0.004174	0.003326	0.012515	0.007269	0.004588	0.004756	0.002418	0.004969	0.004885
	33-th file	0.008535	0.005003	0.004349	0.013606	0.007391	0.005139	0.005212	0.003501	0.005347	0.004885
	34-th file	0.0095	0.005117	0.004349	0.013606	0.007867	0.005969	0.007679	0.003501	0.005347	0.006702
	35-th file	0.010652	0.005862	0.004349	0.01429	0.007867	0.005969	0.007679	0.003501	0.006068	0.00874
	36-th file	0.01234	0.005969	0.004349	0.01429	0.008121	0.005969	0.00775	0.003501	0.006068	0.008789
	37-th file	0.01234	0.005969	0.005125	0.01529	0.008121	0.007125	0.00775	0.003501	0.006068	0.010979
	38-th file	0.012937	0.006847	0.005125	0.01529	0.008456	0.007125	0.00775	0.004128	0.006068	0.010979
	39-th file	0.013447	0.006847	0.005125	0.015979	0.008456	0.007125	0.00775	0.004128	0.006068	0.010979
	40-th file	0.013618	0.007854	0.005429	0.015979	0.008456	0.007125	0.008187	0.004128	0.006068	0.010979
	41-th file	0.013838	0.007854	0.005429	0.015979	0.008456	0.007125	0.008187	0.004128	0.006969	0.011177

Table S2: Byte error rate at average coverage of 4.4x.

		1	2	3	4	5	6	7	8	9	10
Sequencing reads number		1127843	1126623	1128481	1124816	1126739	1127327	1111876	1116384	1114664	1113827
Mean coverage		4.418616	4.413837	4.421116	4.406757	4.414291	4.416595	4.356061	4.373722	4.366984	4.363705
November 2021	0-th file	0	0	0	0	0	0	0	0	0	0
	1-th file	0	0	0	0	0	0	0	0	0	0
	2-th file	0	0	0	0	0	0	0	0	0	0
	3-th file	0	0	0	0	0	0	0	0	0	0
	4-th file	0	0	0	0	0	0	0	0	0	0
	5-th file	0	0	0	0	0	0	0	0	0	0
	6-th file	0	0	0	0	0	0	0	0	0	0
	7-th file	0	0	0	0	0	0	0	0	0	0
	8-th file	0	0	0	0	0	0.00025	0	0	0	0
	9-th file	0	0	0	0	0	0.00025	0	0	0	0
	10-th file	0	0	0	0	0	0.00025	0	0	0	0
	11-th file	0	0	0	0	0	0.00025	0	0	0	0
	12-th file	0	0	0	0	0	0.00025	0	0	0	0
	13-th file	0	0	0	0	0	0.00025	0	0	0	0
	14-th file	0	0	0	0	0	0.00025	0	0	0	0
	15-th file	0	0	0	0	0	0.00025	0	0	0	0
	16-th file	0	0	0	0	0	0.00025	0	0	0	0.00028
	17-th file	0	0	0	0	0	0.00025	0	0	0	0.00028
	18-th file	0	0	0	0	0	0.00025	0	0	0	0.00028
	19-th file	0	0	0	0	0	0.00025	0	0	0	0.00028
	20-th file	0	0	0	0	0	0.00025	0	0	0	0.00028
October 2023	21-th file	0	0	0.002197	0	0.004524	0.001939	0	0	0	0
	22-th file	0.00219	0	0.002197	0	0.004912	0.001939	0.000776	0.00157	0.000973	0.003277
	23-th file	0.00219	0	0.002197	0	0.004912	0.002579	0.000776	0.00157	0.000973	0.003277
	24-th file	0.00219	0	0.002197	0	0.004912	0.002579	0.000825	0.00157	0.000973	0.003277
	25-th file	0.00219	0.000798	0.003267	0	0.005881	0.003392	0.000825	0.00157	0.000973	0.003277
	26-th file	0.00312	0.000798	0.003267	0	0.005881	0.003392	0.000825	0.00157	0.001016	0.003277
	27-th file	0.00312	0.000798	0.003267	0	0.005881	0.003392	0.000825	0.00157	0.001016	0.003277
	28-th file	0.00312	0.001878	0.003267	0	0.006391	0.003392	0.000825	0.00157	0.001016	0.003277
	29-th file	0.00312	0.001878	0.003267	0	0.006391	0.003392	0.000825	0.00157	0.001016	0.003277
	30-th file	0.00312	0.001878	0.003267	0	0.006391	0.003392	0.000825	0.00157	0.001016	0.003277
	31-th file	0.00312	0.001878	0.003267	0	0.006391	0.003392	0.000825	0.00157	0.001016	0.003277
	32-th file	0.00312	0.002598	0.004286	0.002102	0.007341	0.003392	0.002579	0.00157	0.001016	0.003741
	33-th file	0.003884	0.002598	0.004286	0.002102	0.008091	0.003392	0.002579	0.00157	0.001692	0.003741
	34-th file	0.003884	0.002598	0.004286	0.002102	0.008091	0.003392	0.002579	0.00157	0.001744	0.003741
	35-th file	0.003884	0.002598	0.004286	0.002102	0.008091	0.004126	0.002579	0.00157	0.001793	0.003741
	36-th file	0.003884	0.004031	0.004286	0.002102	0.008091	0.004126	0.002579	0.0021	0.001899	0.004467
	37-th file	0.003884	0.004989	0.004286	0.002912	0.008091	0.004126	0.002579	0.0021	0.001899	0.004467
	38-th file	0.003884	0.004989	0.004286	0.002912	0.008091	0.004126	0.002579	0.0021	0.001899	0.004467
	39-th file	0.003884	0.004989	0.004286	0.002912	0.008091	0.004126	0.002579	0.0021	0.001899	0.004719
	40-th file	0.003884	0.004989	0.004286	0.002912	0.008091	0.004126	0.002579	0.002487	0.002988	0.005723
	41-th file	0.003884	0.004989	0.004286	0.002912	0.008091	0.004126	0.002579	0.002487	0.002988	0.005723

Table S3: Byte error rate at average coverage of 4.6x.

		1	2	3	4	5	6	7	8	9	10
Sequencing reads number		1180469	1179741	1180747	1179358	1181080	1180194	1180747	1179358	1180243	1177762
Mean coverage		4.624792	4.62194	4.625881	4.62044	4.62719	4.623715	4.625881	4.61044	4.62391	4.614187
	0-th file	0	0	0	0	0.0002052	0	0	0	0.001465	0
	1-th file	0	0	0	0	0.0002052	0	0	0	0.001465	0
	2-th file	0	0	0	0	0.0002052	0	0	0	0.001465	0
	3-th file	0	0	0	0	0.0002052	0	0	0	0.001465	0
	4-th file	0	0	0	0	0.0002052	0	0	0	0.001465	0
	5-th file	0	0	0	0	0.0002052	0	0	0	0.001465	0
	6-th file	0	0	0	0	0.0002052	0	0	0	0.001465	0
	7-th file	0	0	0	0	0.0002052	0	0	0	0.001465	0
	8-th file	0	0	0	0	0.0002052	0	0	0	0.001465	0
	9-th file	0	0	0	0	0.0002052	0	0	0	0.001465	0
November 2021	10-th file	0	0	0	0	0.0002052	0	0	0	0.001465	0
	11-th file	0	0	0	0	0.0002052	0	0	0	0.001465	0
	12-th file	0	0	0	0	0.0002052	0	0	0	0.001465	0
	13-th file	0	0	0	0	0.0002052	0	0	0	0.001465	0
	14-th file	0	0	0	0	0.0002052	0	0	0	0.001465	0
	15-th file	0	0	0	0	0.0002052	0	0	0	0.001465	0
	16-th file	0	0	0	0	0.0002052	0	0	0	0.001465	0
	17-th file	0	0	0	0	0.0002052	0	0	0	0.001465	0
	18-th file	0	0	0	0	0.0002052	0	0	0	0.001465	0
	19-th file	0	0	0	0	0.0002052	0	0	0	0.001465	0
	20-th file	0	0	0	0	0.0002052	0	0	0	0.001465	0
	21-th file	0	0.000137	0	0.002327	0	0	0	0.001231	0	0
	22-th file	0	0.000137	0.000741	0.004737	0	0	0.000741	0.00341	0	0
	23-th file	0	0.000137	0.000741	0.004737	0	0	0.000741	0.00341	0	0
	24-th file	0	0.000137	0.000741	0.004737	0	0	0.000741	0.00341	0	0
	25-th file	0	0.000137	0.000741	0.004737	0	0	0.000741	0.00341	0	0
	26-th file	0	0.000137	0.000741	0.004737	0	0	0.000741	0.00341	0	0
	27-th file	0	0.000137	0.000741	0.004737	0	0	0.000741	0.00341	0	0
	28-th file	0	0.000137	0.000741	0.004737	0	0	0.000741	0.00341	0	0
	29-th file	0	0.000137	0.000741	0.004737	0	0	0.000741	0.00341	0	0
	30-th file	0	0.000137	0.000741	0.004737	0	0	0.000741	0.00341	0	0
October 2023	31-th file	0	0.000137	0.000741	0.004737	0	0	0.000741	0.00341	0	0
	32-th file	0	0.000137	0.001574	0.00574	0	0	0.000833	0.004023	0	0.000741
	33-th file	0	0.000137	0.001574	0.00574	0	0	0.000833	0.004023	0	0.000741
	34-th file	0.001019	0.001038	0.001574	0.00574	0	0.000852	0.000833	0.004023	0	0.000741
	35-th file	0.001019	0.001038	0.001574	0.00574	0	0.000852	0.000833	0.004023	0	0.000741
	36-th file	0.001019	0.001038	0.001574	0.00574	0	0.000852	0.000833	0.004023	0	0.000741
	37-th file	0.001019	0.001038	0.001574	0.00574	0	0.000852	0.000833	0.004023	0	0.000741
	38-th file	0.001019	0.001038	0.001574	0.00574	0	0.000852	0.000833	0.004023	0	0.000741
	39-th file	0.001019	0.001038	0.001574	0.00574	0	0.00106	0.000833	0.004023	0	0.000741
	40-th file	0.001019	0.001038	0.001574	0.00574	0	0.00106	0.000833	0.004023	0	0.000741
	41-th file	0.001019	0.001038	0.001574	0.00574	0	0.00106	0.000833	0.004023	0	0.000741

Table S4: Byte error rate at average coverage of 4.8x.

		1	2	3	4	5	6	7	8	9	10
Sequencing reads number		1200056	1212942	1211622	1213817	1231793	1232207	1230462	1225255	1223899	1226132
Mean coverage		4.701528	4.752014	4.746842	4.755442	4.825867	4.827493	4.820653	4.800253	4.794941	4.803689
	0-th file	0	0	0	0	0	0	0	0	0	0
	1-th file	0	0	0	0	0	0	0	0	0	0
	2-th file	0	0	0	0	0	0	0	0	0	0
	3-th file	0	0	0	0	0	0	0	0	0	0
	4-th file	0	0	0	0	0	0	0	0	0	0
	5-th file	0	0	0	0	0	0	0	0	0	0
	6-th file	0	0	0	0	0	0	0	0	0	0
	7-th file	0	0	0	0	0	0	0	0	0	0
	8-th file	0	0	0	0	0	0	0	0	0	0
	9-th file	0	0	0	0	0	0	0	0	0	0
November 2021	10-th file	0	0	0	0	0	0	0	0	0	0
	11-th file	0	0	0	0	0	0	0	0	0	0
	12-th file	0	0	0	0	0	0	0	0	0	0
	13-th file	0	0	0	0	0	0	0	0	0	0
	14-th file	0	0	0	0	0	0	0	0	0	0
	15-th file	0	0	0	0	0	0	0	0	0	0
	16-th file	0	0	0	0	0	0	0	0	0	0
	17-th file	0	0	0	0	0	0	0	0	0	0
	18-th file	0	0	0	0	0	0	0	0	0	0
	19-th file	0	0	0	0	0	0	0	0	0	0
	20-th file	0	0	0	0	0	0	0	0	0	0
	21-th file	0	0	0	0	0	0	0	0	0	0
	22-th file	0	0	0	0.001581	0	0	0	0	0	0
	23-th file	0	0	0	0.001581	0	0	0	0	0	0
	24-th file	0	0	0	0.001581	0	0	0	0	0	0
	25-th file	0	0	0	0.001581	0	0	0	0	0	0
	26-th file	0	0	0	0.001581	0	0	0	0	0	0
	27-th file	0	0	0	0.001581	0	0	0	0	0	0
	28-th file	0	0	0	0.001581	0	0	0	0	0	0
	29-th file	0	0	0	0.001581	0	0	0	0	0	0
	30-th file	0	0	0	0.001581	0	0	0	0	0	0
October 2023	31-th file	0	0	0	0.001581	0	0	0	0	0	0
	32-th file	0	0	0	0.001581	0	0	0	0	0	0
	33-th file	0	0	0	0.001581	0	0	0	0	0	0
	34-th file	0	0	0	0.001581	0	0	0	0	0.000266	0
	35-th file	0	0	0.00084	0.001581	0	0	0	0	0.000266	0
	36-th file	0	0	0.00084	0.001581	0	0	0	0	0.000266	0
	37-th file	0	0	0.00084	0.001581	0	0	0	0	0.000266	0
	38-th file	0	0	0.00084	0.001581	0	0	0	0	0.000266	0
	39-th file	0	0	0.00084	0.001581	0	0	0	0	0.000266	0
	40-th file	0	0	0.00084	0.001581	0	0	0	0	0.000266	0
	41-th file	0	0	0.00084	0.001581	0	0	0	0	0.000266	0

Table S5: Encoding results for frame-by-frame surveillance images.

	frame-by-frame surveillance files	1	2	3	4	5	6	7	8	9	10	11	12	13	14	15	16	17	18	19	20	Total
	Data size (MB)	6.2	6.2	6.2	6.2	6.2	6.2	6.2	6.2	6.2	6.2	6.2	6.2	6.2	6.2	6.2	6.2	6.2	6.2	6.2	6.2	124.42
$r(\frac{b}{n})$	With DNA Palette code	1.52	1.94	1.94	2.34	2.03	1.98	2.32	1.91	2.31	3.50	1.81	2.51	2.90	1.82	1.83	2.87	2.03	2.00	2.12	2.20	2.12
	Without DNA Palette code	1.52	1.52	1.52	1.52	1.52	1.52	1.52	1.52	1.52	1.52	1.52	1.52	1.52	1.52	1.52	1.52	1.52	1.52	1.52	1.52	1.52

Supplementary Data

The dataset used in our experiments is available in https://doi.org/10.6084/m9.figshare.25567185.v2.

Data S1: Dataset of the MRI files (DICOM) stored in our wet experiment.

Data S2: Dataset of the Earth plasmasphere observations acquired by the extreme ultraviolet camera onboard the Chang'E-3 lander.

Data S3: Dataset of the threshold method of the microwave radiometer's day and winter brightness temperature difference to extract the Greenland Ice Sheet surface melt from the downscaling results, and obtain the 0.05° daily melt results of the Greenland Ice Sheet surface in 1985, 2000, and 2015.

Supplementary movies

The end to end presentation of our encoding, decoding and testing processes is available in https://doi.org/10.6084/m9.figshare.25567299.v2.

Movie S1: Full demonstration of the DNA palette encoding process on the MRI dataset. The video shows the encoding command lines. The output (codewords) is codeword.txt.

Movie S2: Full demonstration of the decoding process of the DNA palette code on the MRI dataset. The decoding output is a DICOM dataset for medical diagnosis.

Movie S3: Full demonstration of the DNA palette testing process. The test first encodes a randomly generated information sequence, then inputs the encoded sequence into a simulated DNA channel, and finally decodes the error-prone DNA channel output.
